# Misspecification of Cox regression models with composite endpoints

**DOI:** 10.1002/sim.5436

**Published:** 2012-06-27

**Authors:** Longyang Wu, Richard J Cook

**Affiliations:** Department of Statistics and Actuarial Science, University of WaterlooWaterloo, Ontario, N2L 3G1, Canada

**Keywords:** copula, composite endpoint, Cox regression, model misspecification, randomized clinical trial

## Abstract

Researchers routinely adopt composite endpoints in multicenter randomized trials designed to evaluate the effect of experimental interventions in cardiovascular disease, diabetes, and cancer. Despite their widespread use, relatively little attention has been paid to the statistical properties of estimators of treatment effect based on composite endpoints. We consider this here in the context of multivariate models for time to event data in which copula functions link marginal distributions with a proportional hazards structure. We then examine the asymptotic and empirical properties of the estimator of treatment effect arising from a Cox regression model for the time to the first event. We point out that even when the treatment effect is the same for the component events, the limiting value of the estimator based on the composite endpoint is usually inconsistent for this common value. We find that in this context the limiting value is determined by the degree of association between the events, the stochastic ordering of events, and the censoring distribution. Within the framework adopted, marginal methods for the analysis of multivariate failure time data yield consistent estimators of treatment effect and are therefore preferred. We illustrate the methods by application to a recent asthma study. Copyright © 2012 John Wiley & Sons, Ltd.

## 1 Introduction

Many diseases put individuals at elevated risk for a multitude of adverse clinical events, and researchers routinely design randomized clinical trials to evaluate the effectiveness of experimental interventions for the prevention of these events. Trials in cardiology, for example, record times of events such as non-fatal myocardial infaction, non-fatal cardiac arrest, and cardiovascular death [1]. In cerebrovascular disease, patients with carotid stenosis can be treated with medical therapy or surgery, and trials evaluating their relative effectiveness may record endpoints such as strokes ipsilateral to the surgical site, contralateral strokes, and death [2]. In oncology, researchers often design trials to study treatment effects on disease progression and death [3], but palliative trials of patients with skeletal metastases may be directed at preventing skeletal complications including vertebral and non-vertebral fractures, bone pain, and the need for surgery to repair bone [4]. In these and many other settings, although interest lies in preventing each of the respective events, it is generally infeasible to conduct studies to answer questions about each component.

When one type of event is of greater clinical importance than others, it can be chosen as the basis of the primary treatment comparison, and effects on other types of events can then be assessed through secondary analyses. When two or more events are of comparable importance, co-primary endpoints can be specified, but tests of hypotheses must typically control the experimental type I error rate through multiple comparison procedures 5–7; these make decision analyses more complex. A seemingly simple alternative strategy is to adopt a so-called composite event 8,9 that is said to have occurred if any one of a set of component events occurs. The time of the composite event is therefore the minimum of the times of all component events.

There are several additional reasons investigators may consider the use of composite endpoints in clinical trials. In studies involving a time-to-event analysis, the use of a composite endpoint will mean that more events will be observed than would be observed for any particular component. If the same clinically important effect is specified for the composite endpoint and one of its components, this increased event rate will translate into greater power for tests of treatment effects; at the design stage this translates to a reduction in the required number of subjects or duration of follow-up 9–11. Composite endpoints are routinely adopted through the introduction of one or more less serious events, however, which presumably warrants revising the clinically important effect of interest. Moreover, we show later that with models featuring a high degree of structure, model assumptions may not even be compatible for the composite endpoint and one of its components.

In time-to-event analyses, interest may lie in the effect of an experimental treatment versus standard care on the risk of a non-fatal event. This is a common framework in trials of patients with advanced diseases where interest lies in improving quality of life through the prevention of complications. In such settings, individuals are at considerable risk of death and a competing risks problem arises. Investigators often deal with this by adopting a composite endpoint based on the time to the minimum of the non-fatal event of interest and death 12,13. This strategy leads to an ‘event-free survival’ analysis that is particularly common in cancer where progression-free survival is routinely adopted as a primary endpoint [14]. In palliative trials, however, a treatment may not be expected to have an effect of survival, and if a non-negligible proportion of individuals die before experiencing the clinical event of interest, this analysis can lead to a serious underestimation of the effect of the treatment 10,15.

Recommendations are available in the literature on how to design trials, analyze resultant data, and report findings when composite endpoints are to be used 10–12,16. The main recommendations include that (i) individual components should have similar frequency of occurrence, (ii) the treatment should have a similar effect on all components, (iii) individual components should have similar importance to patients, (iv) data from all components should be collected until the end of trial, and (v) individual components should be analyzed and reported separately as secondary endpoints. The first three recommendations have face validity and seem geared towards helping ensure that conclusions regarding treatment effects on the composite endpoint have some relation to treatment effects on the component endpoints, thus helping in the interpretation of results. The collection of data on the occurrence of the component endpoints until the end of the trial facilitates separate assessment of treatment effects on each of the component endpoints. This means the consistency of findings across components can be empirically assessed.

The aforementioned issues have been actively debated in the medical literature 11,16–19, but there has been relatively little formal statistical investigation of these points. In this paper, we discuss statistical considerations related to composite endpoint analyses and use the recommendations to guide the investigation. Because the Cox regression model is routinely adopted for the analysis of composite endpoints in clinical trials [12], we consider it here and point out important issues regarding model specification and interpretation. We formulate multivariate failure time models with proportional hazards for the marginal distributions that may be used to reflect the settings where composite endpoints are most reasonable according to the current guidelines. We study the asymptotic and empirical properties of estimators arising from a composite endpoint analysis. We also explore the utility of marginal methods based on multivariate failure time data [20]. We argue that the belief that composite endpoints provide an overall measure of the effect of treatment is overly simplistic, and a thoughtful interpretation of intervention effects based on composite endpoints alone is difficult. Their use as a primary basis for treatment comparison in clinical trials therefore warrants careful consideration.

The remainder of this paper is organized as follows. In Section 2, we construct bivariate failure time distributions for which the marginal distributions have proportional hazards between two treatment groups. We then derive the distribution for the time to the first event and show that it does not typically feature proportional hazards across the two treatment groups. We use large sample theory for misspecified models to derive the limiting value of the log hazard ratio from a naive Cox model, and empirical studies demonstrate finite sample properties which are in close alignment with the theory. An alternative approach to synthesizing data over component events is to conduct a global analysis on the basis of the marginal methods of Wei *et al.* 1989; we explore this in Section 3. An application to a recently completed asthma management study illustrates the various methods in Section 4, and we make the concluding remarks in Section 5.

## 2 Multivariate failure time distributions via copula functions

### 2.1 Construction of joint distributions based on copula functions

If (*U*_1_, *U*_2_) ′ is a bivariate random variable with standard uniform margins on [0,1], a two-dimensional copula function can be defined as 

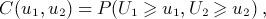
(1)
[21]. If there exists a convex decreasing function 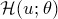 such that 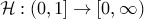 and 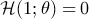, and if the copula function can be written as 

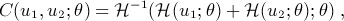

then copula belongs to the *Archimedean family*; the univariate function 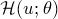 is called the *generator* for the copula [22]. Suppose (*U*_*i*1_, *U*_*i*2_) ′ and (*U*_*j*1_, *U*_*j*2_) ′ are two random variables drawn from the joint distribution 1. A common measure of the association between *U*_1_ and *U*_2_ is Kendall's *τ*, defined as 

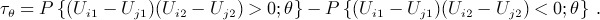

where we write *τ*_*θ*_ to make the relation between *θ* and *τ* explicit.

For Archimedean copulas, this can be written as 

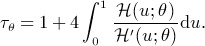


Copula functions have received considerable attention in the statistical literature in the past few years because they offer a convenient and attractive way of linking two marginal distributions to create a joint survival function [23]. Suppose *T*_1_ and *T*_2_ are a pair of non-negative random variables with respective survivor functions 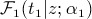 and 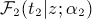 given a covariate *z*. If we let 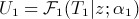 and 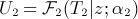 where *α*_*k*_ indexes the marginal distribution for *T*_*k*_ | *z*, then *U*_*k*_ ∼ UNIF(0,1), *k* = 1,2. We can define the bivariate ‘survival’ distribution function of (*U*_1_,*U*_2_) through a copula as in 1 and obtain a joint survivor function for (*T*_1_,*T*_2_) ′ given *Z* as 

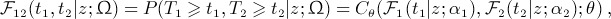
(2) where Ω = (*α*′, *θ*)′ with *α* = (*α*′, *α*′)′. Because Kendall's *τ* is invariant to monotonic increasing or decreasing transformations [21], it can be interpreted as a measure of association of the transformed variables (*T*_1_,*T*_2_) ′ given *Z*. The use of a copula function to define the joint distribution of (*T*_1_,*T*_2_)|*z* is particularly appealing because one can specify the marginal distributions to have a proportional hazards form; this is not typically possible for joint distributions induced by random effects or intensity-based analyses.

If a composite endpoint analysis is planned, it would be based on modeling the random variable *T* = min(*T*_1_, *T*_2_), which has survival, density, and hazard function conditional on *Z*, given by 

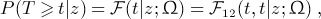
(3)

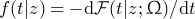
, and 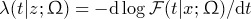, respectively. Suppose *Z* is a binary indicator where *Z* = 1 for individuals in a treatment group and *Z* = 0 otherwise. A key point is that the hazard ratio *λ*(*t*|*z* = 1; Ω) / *h*(*t*|*z* = 0; Ω) is not, in general, independent of time. As a result, even if the marginal distributions feature proportional hazards, the model for the composite endpoint will typically not. We study this point further in the next four settings for three different Archimedean copulas and the case of independent components.

#### 2.1.1 Composite endpoint analysis based on a Clayton copula

The Clayton copula 1978 is a member of the Archimedean family with generator 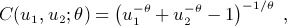, 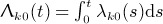 and copula function 

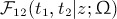
(4) with *θ* ≥ −1. Kendall's *τ* is then given by *τ*_*θ*_ = *θ* / (*θ* + 2), which can be seen to vary over [−1, 1].

Consider the joint distribution of (*T*_1_, *T*_2_)|*Z* in which the marginal distribution for *T*_*k*_|*Z*, 1,2 features proportional hazards; so *λ*_*k*_(*t*|*z*) = *λ*_*k*0_(*t*) exp(*β*_*k*_*z*) with Λ_*k*_(*t*|*z*) = Λ_*k*0_(*t*) exp(*β*_*k*_*z*) where 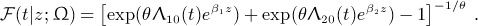, *k* = 1,2. If the joint survivor function 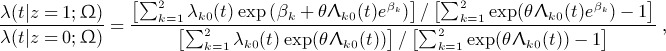 is determined by the Clayton copula through 5, by 6 the survivor function of the failure time *T* = min(*T*_1_, *T*_2_) given *z* is 

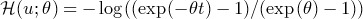
(5) Hence, the hazard ratio for the treatment versus control groups for the composite endpoint is 

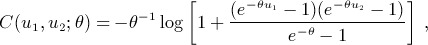
(6) which is not invariant with respect to time in general.

**Figure 1:**
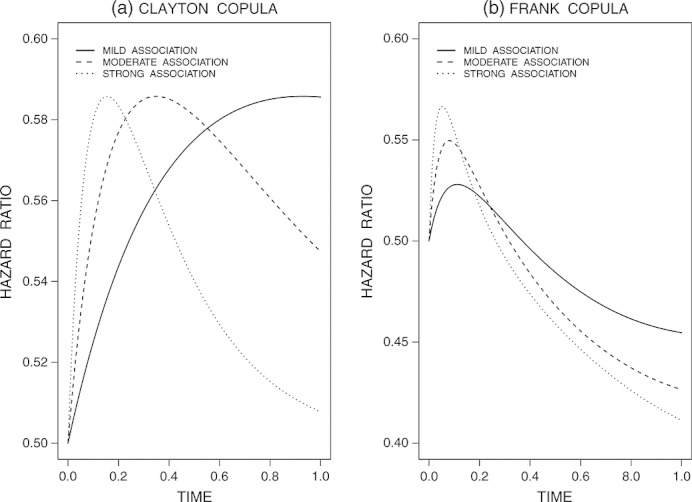
Plots of the hazard ratio over the time interval [0,1] for the composite endpoint model implied by the Clayton copula (panel (a)) and Frank copula (panel (b)) with marginal exponential distributions with λ_1_ = λ_2_ = log 10 and exp(β_1_) = exp(β_2_) = exp(β) = 0.50 and mild (*τ*_θ_ = 0.20), moderate (τ_θ_ = 0.40), and strong (τ_θ_ = 0.60) associations.

To gain some insight into this function, suppose the marginal distributions are exponential with common baseline hazards of *λ*_10_(*t*) = *λ*_20_(*t*) = *λ* = log 10 so that the probability of a type *k* event occurring before *t* = 1 is 0.90 for a control subject (i.e., *P*(*T*_*k*_ < 1 | *Z* = 0) = 0.90). Further suppose that a common hazard ratio of 0.50 holds for the two margins (i.e., exp(*β*_1_) = exp(*β*_2_) = 0.50). This setting is consistent with the recommendations that the component events occur with comparable frequency because *P*(*T*_1_ < *T*_2_ | *Z*) = 0.5, and have comparable treatment effects (*β*_1_ = *β*_2_). Figure 1(a) contains a plot of the hazard ratio 9 over the time interval [0,1] for models with mild (*τ*_*θ*_ = 0.2), moderate (*τ*_*θ*_ = 0.40), and strong (*τ*_*θ*_ = 0.60) association. As can be seen, even when the treatment effects are the same for the two component endpoints, there can be non-negligible variation in the hazard ratio over time, and within this family of models, the nature of this variation depends on the strength of the association between the two failure times.

#### 2.1.2 Composite endpoint analysis based on a Frank copula

The generator for the Frank copula [25] is 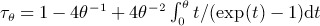, and the resulting copula function is 

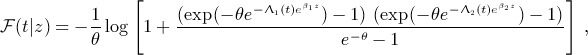
 where 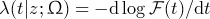; Kendall's τ is then 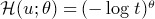. If we adopt the same marginal distributions as before, the survivor function for the composite endpoint is 

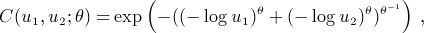
 but because 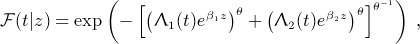, the hazard ratio λ(*t*|*z* = 1; Ω) / λ(*t*|*z* = 0; Ω) has a complicated form. Figure 1(b) contains a plot of this hazard ratio over [0,1], and as in the case of the Clayton copula, there is considerable variation in this ratio over time.

#### 2.1.3 Composite endpoint analysis based on a Gumbel–Hougaard copula

The generator for the 26 copula is 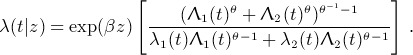 giving 

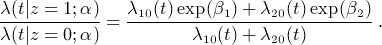

for θ ≥ 1; Kendall's τ is given by τ_θ_ = (θ − 1) / θ. The corresponding survivor function for the composite endpoint is 

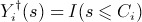
 and if β_1_ = β_2_ = β, the hazard is 

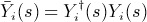

Interestingly, the hazard ratio in this case is exp(β), which means that the proportional hazards model for the composite endpoint is compatible with a proportional hazards model for the margins. If the hazard ratio is in fact common for the component endpoints, then a consistent estimator will be obtained for this common effect on the basis of a Cox model for the composite endpoint.

#### 2.1.4 Composite endpoint analysis with independent components

Here, we consider the setting where the component failure times are independent; a special case of τ_θ_ = 0 for the joint models in Sections 2.1.1–2.1.3. In this case, the hazard ratio for the composite endpoint analysis reduces to 

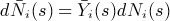


With nonhomogeneous hazards, it is apparent that the composite endpoint analysis is only compatible with a proportional hazards assumption if either (A.1) β_1_ = β_2_ = β or (A.2) λ_10_(*t*) = λ_20_(*t*). If β_1_ = β_2_ = β, then a consistent estimate of this common effect is obtained in a composite endpoint analysis. If β_1_ ≠ β_2_ but the hazard functions are identical, the multiplicative effect is (exp(β_1_) + exp(β_2_)) / 2. If assumptions A.1 and A.2 do not hold, then the ratio is a complicated time varying function of the baseline hazards and respective treatment effects.

### 2.2 Misspecification of the Cox model with composite endpoints

The previous section demonstrated that the composite endpoint analysis is typically based on a misspecified Cox regression model if the marginal distributions satisfy the proportional hazards assumption. In this section, we investigate the frequency properties of estimators from a composite endpoint analysis when the component endpoints are associated through a copula function.

Let *T*_*i*_ = min(*T*_*i*1_,*T*_*i*2_) denote the time of the composite endpoint for individual *i* in a sample of size *m*. Let {*N*_*i*_(*s*),*s* < 0} denote the counting process for subject *i*, which indicates the occurrence of the composite endpoint, so that *dN*_*i*_(*s*) = 1 if *T*_*i*_ = *s* and is zero otherwise. Suppose that it is planned to follow all subjects over the interval (0,*C*^†^] but that subjects may be lost to follow-up or withdraw from the study prematurely. Let *W*_*i*_ represent the withdrawal time for subject *i* and *C*_*i*_ = min(*W*_*i*_,*C*^†^) denote their right censoring time. Let *Y*_*i*_(*s*) = *I*(*s* ≤ *T*_*i*_) indicate whether subject *i* is at risk of the composite endpoint at time *s*, 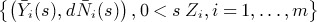 indicate whether they are under observation at time *s*, and 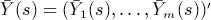 indicate whether they are event free and under observation. The observable counting process for the response is then based on 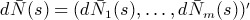 for subject *i*. The data for a sample of size *m* then consist of 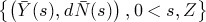, which if we let 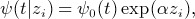, 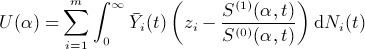 and *Z* = (*Z*_1_, …,*Z*_*m*_)′, we may write more compactly as 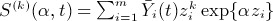.

The Cox model is widely used in the analysis of composite endpoints 1972 to estimate the relative hazard where we assume the hazard function for *T*_*i*_|*z*_*i*_ to have the form 

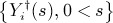
 where *ψ*_0_(*t*) is a non-negative baseline hazard function corresponding to the control group and *z*_*i*_ is the treatment covariate for individual *i*, *i* = 1, …,*m*. The treatment effect *α* can be estimated using the maximum partial likelihood [28] by solving 

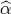
 where 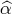, *k* = 0,1.

If 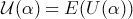 is independent of {*N*_*i*_(*s*),0 < *s*} given *Z*_*i*_ and if 16 is correctly specified, then 17 has expectation zero and the solution 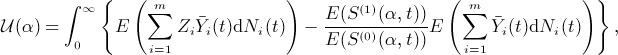 is consistent for the true value, *α*. In the independence case, this true value is β if the treatment effect is common (i.e., under A.1 β = β_1_ = β_2_) or *α* = log(exp(β_1_) + exp(β_2_)) / 2 if the baseline hazard functions are the same (i.e, under A.2). More generally, however, 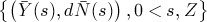 is consistent for α*, the solution to expected score function 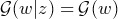 given by 

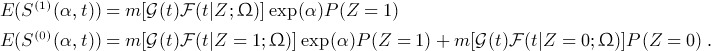
 where the expectation *E* is with respect to the true model for 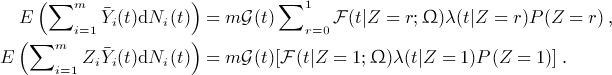 29–31. By using the true model based on 6 and assuming independent censoring for the withdrawal time *W*_*i*_ with survival distribution 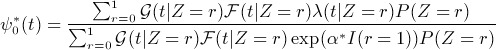, these expectations can be obtained as follows: 

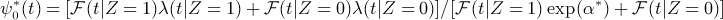
 Likewise, 

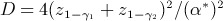


To illustrate the bias resulting from a composite endpoint analysis, consider a randomized clinical trial in which subjects are to be followed over the interval (0,*C*^†^] where *C*^†^ = 1. Let *Z* = 1 for treated subjects and *Z* = 0 for control subjects and suppose *P*(*Z* = 1) = 1 − *P*(*Z* = 0) = 0.5. We set β_1_ = β_2_ = β = log 0.80 to consider the case compatible with the current recommendations on the use of composite endpoints. We set λ_1_ and λ_2_ so that (i) *P*(*T*_1_ < *T*_2_|*Z* = 0) = *p*_1_ equals a desired probability that the type 1 event occurs before the type 2 event among control subjects and that (ii) *P*(*C*^†^ < *T*) = *π*_*A*_ satisfies the administrative censoring rate for the composite endpoint among all subjects, where *π*_*A*_ = 0.20. Finally, suppose subjects may withdraw from the study early, and let *W* have an exponential distribution with rate *ρ* such that *P*(*C* < *T*) = *π*, where *P*(*C* < *T*) = *E*_*Z*_[*P*(*W* < *T* < *C*^†^|*Z*) + *P*(*C*^†^ < *T*|*Z*)] and *π* is the overall censoring rate set to *π* = 0.20, 0.40, 0.60, and 0.80.

**Figure 2:**
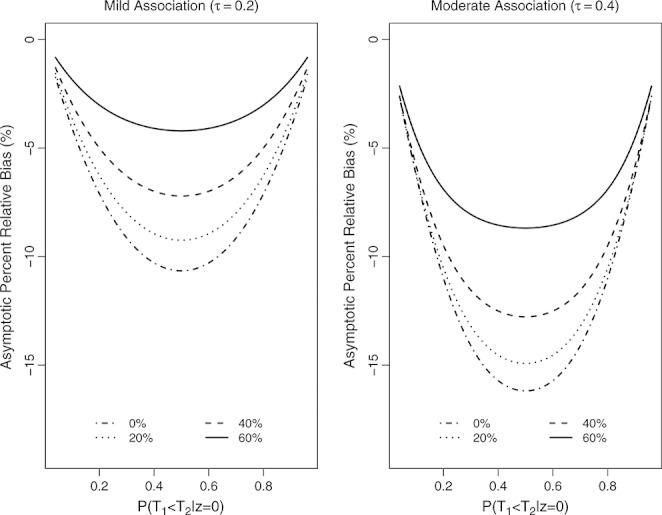
Asymptotic percent relative bias (100 (α* − β) / β) of Cox regression coefficient of treatment effect from composite endpoint analysis when bivariate failure times are generated by a Clayton copula; exponential margins, 20% administrative censoring (*π*_*A*_ = 0.20), 50:50 randomization, exp(β_1_) = exp(β_2_) = 0.80, and four different degrees of additional random censoring (none, 20%, 40%, and 60%).

Figure 2 shows the limiting percent relative bias ( 100(*α*^∗^ − β) / β) of the treatment coefficient from a composite endpoint analysis when the data are generated by a Clayton copula with mild (τ = 0.20) and moderate (τ = 0.40) association. We plotted this relative bias against *P*(*T*_1_ < *T*_2_|*Z* = 0) = *p*_1_, and interestingly, the bias is greatest when *p*_1_ = 0.50 but decreases as this probability approaches zero or one. In either of the extreme cases (*p*_1_ = 0 or *p*_1_ = 1), the composite endpoint coincides with the occurrence of a single endpoint, and a consistent estimate of the common treatment effect is obtained. Note that the bias (α* − β) is positive, and hence, the limiting value of the treatment effect is more conservative than the true common value for each of the components. This means that the estimated value would, on average, under-represent the magnitude of the treatment effect on either component, a conclusion in line with the findings of 10,15. Moreover, we note that the common event rate and the common treatment effect are precisely the setting where composite endpoints are recommended for use 10,11,12,16. The plots also reveal the sensitivity of the limiting value to the degree of random censoring; the higher the censoring rate, the smaller the asymptotic bias. This highlights an important point that the limiting value of an estimator from a misspecified failure time model is highly sensitive to the censoring distribution even under independent censoring. By comparing the left and right panels in Figure 2, it is also apparent that the asymptotic bias is dependent on the degree of association between *T*_1_ and *T*_2_; the greater the association, the greater the asymptotic bias. This makes sense because when the event times are independent, consistent estimates should be obtained because assumptions A.1 and A.2 of Section 2.1.4 are satisfied.

Although of secondary interest, one can also show that 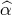, 0 < *t* < *C*^†^, is consistent for 

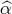
 which when *P*(*Z* = 1) = 0.5 and the censoring distribution is the same in the two groups reduces to 

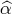


### 2.3 Simulation studies involving composite endpoints

#### 2.3.1 Simulation design

Here, we simulate data from 5 to examine the empirical performance of estimators for finite samples. We assume that given *Z*, *T*_*k*_ has an exponential distribution with hazard λ_*k*_exp(β_*k*_*Z*), *k* = 1,2, and model the association between *T*_1_ and *T*_2_ through a Clayton copula. We let *T* = min(*T*_1_,*T*_2_) denote the time of the composite endpoint as before. We suppose interest lies in following subjects over (0,1]. As in the previous section, we determined the parameters λ_1_ and λ_2_ to satisfy the constraints *P*(*T*_1_ < *T*_2_|*Z* = 0) = *p*_1_, where *p*_1_ = 0.25, *P*(*C*^†^ < *T*) = *π*_*A*_, and we set the administrative censoring rate to *π*_*A*_ = 0.20. We also incorporated random loss to follow-up with an exponential withdrawal time giving a net censoring rate of *π* = 0.20, 0.40, 0.60, and 0.80 subject to the constraint *π*_*A*_ ≤ *π*.

For each parameter configuration, we derived the sample size for the composite endpoint analysis to achieve a prespecified power under the assumption that the Cox model in 16 holds. Therneau and Grambsch (2000) show that the required number of events is 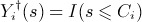, where *z*_*q*_ is the *q*th quantile of the standard Normal distribution, *γ*_1_ is the type I error for a one-sided test, 1 − *γ*_2_ is the power, and α* is the limiting value of treatment effect estimate obtained from 16. We focus on two-sided tests at the 5% significance level (*γ*_1_ = 0.05) and sample sizes to achieve 80% power (*γ*_2_ = 0.20). We calculated the required number of subjects as *m* = *D* / *P*(*T* < *C*). In all simulation studies, we considered both equal treatment effects (β_1_ = β_2_ = β = −.223) and unequal treatment effects (β_1_ = −.223 and β_2_ = 0). For each parameter configuration, we generated 2000 replicates. We report the mean of the 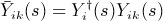 estimates, the empirical standard error (ESE), the average model-based standard error (*ASE*_1_), and the average robust standard error (*ASE*_2_). We also reported the empirical coverage probability (ECP ^∗^%) of nominal 95% CIs for *α*^∗^ based on robust standard errors and the empirical coverage probability of these intervals for β_1_ (ECP%). The last column contains the empirical power (EP%) of a Wald test of the null hypothesis of no treatment effect.

#### 2.3.2 Composite endpoints with dependent components

Table 1 contains the simulation results with dependent component times given by τ = 0.40. The results for equal treatment effects are given in the top half of the table that we comment on first. The fourth column contains *α*^∗^, the limiting value of the estimator from the misspecified Cox model in 16. The fact that these values are all smaller in absolute value than the true common effects reveals the conservative nature of this limiting value, as already discussed in relation to Figure 2; the dependence of the limiting value on the degree of censoring is also apparent. This limiting value was used to derive the sample size (*m*) in the third column. The average estimator from the fitted Cox models reported in the fifth column closely approximates the limiting value. There is also close agreement between the empirical, average model-based, and average robust standard errors. The empirical coverage probabilities of the robust 95% CIs are very close to the nominal levels, and the empirical power is in good agreement with the nominal power of 80%. It is worth emphasizing that the empirical coverage probability is computed for the parameter α*, not the common β; for this latter parameter, the coverage rates are considerably lower.

**Table 1:** Frequency properties of estimators of treatment effect based on a composite endpoint with components arising from a Clayton copula: *p*_1_ = *P*(*T*_1_ < *T*_2_|*z* = 0) = 0.25, β_1_ = −0.223, and τ = 0.4.

*π*_A_	*π*	*m*	α^*^	AVE( 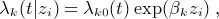 )	ESE	ASE_1_	ASE_2_	ECP^*^%	ECP%	EP%
			*Common treatment effect: β*_2_ = −0.223										
0.2	0.2	816	−0.195	−0.195	0.077	0.079	0.078	95.1	94.1	81.5
	0.4	1071	−0.196	−0.197	0.078	0.079	0.079	95.4	94.3	80.0
	0.6	1557	−0.199	−0.201	0.080	0.081	0.080	94.8	93.8	80.5
	0.8	2908	−0.206	−0.207	0.085	0.083	0.083	94.4	94.5	79.4
0.4	0.4	1076	−0.196	−0.197	0.079	0.079	0.079	95.1	93.1	80.4
	0.6	1557	−0.199	−0.201	0.081	0.080	0.080	94.7	93.6	79.8
	0.8	2907	−0.206	−0.208	0.084	0.083	0.083	95.5	95.0	78.8
0.6	0.6	1522	−0.202	−0.201	0.082	0.081	0.081	94.9	94.3	79.0
	0.8	2886	−0.207	−0.208	0.083	0.084	0.084	95.9	95.2	80.0
0.8	0.8	2779	−0.211	−0.208	0.087	0.085	0.085	94.8	94.1	78.5
			*Different treatment effects: β*_2_ = 0										
0.2	0.2	21743	−0.038	−0.038	0.015	0.015	0.015	94.9	0.0	78.4
	0.4	23103	−0.042	−0.042	0.017	0.017	0.017	94.9	0.0	79.4
	0.6	26037	−0.049	−0.049	0.019	0.020	0.020	95.5	0.0	79.5
	0.8	36581	−0.058	−0.058	0.024	0.023	0.023	94.2	0.0	79.3
0.4	0.4	19221	−0.046	−0.046	0.019	0.019	0.019	94.0	0.0	79.9
	0.6	24084	−0.051	−0.051	0.020	0.020	0.020	95.1	0.0	80.1
	0.8	36376	−0.058	−0.059	0.023	0.023	0.023	94.9	0.0	80.4
0.6	0.6	20656	−0.055	−0.055	0.022	0.022	0.022	94.9	0.0	81.8
	0.8	34960	−0.059	−0.060	0.024	0.024	0.024	95.0	0.0	80.5
0.8	0.8	30990	−0.063	−0.064	0.025	0.025	0.025	95.4	0.0	81.4

*π*_A_ = *P*(*C*^†^ < *T*) is the administrative censoring rate, *π* = *P*(*C*^†^ < *T*) is the net censoring rate, ESE is the empirical standard error, ASE_1_ is the average model-based standard error, ASE_2_ is the average robust standard error, ECP^*^% is the empirical coverage probability for α^*^ of nominal 95% CIs using the robust standard error, ECP% is the empirical coverage probability for β_1_ of nominal 95% CIs using the robust standard error, and EP% is the empirical power of a Wald test of *H*_0_ : *α* = 0 based on the robust standard error.

In the bottom half of Table 1, we reported the results for the case β_1_ ≠ β_2_, where α* is considerably smaller than β_1_. This smaller limiting value leads to considerably larger sample sizes to achieve the desired power. Again, however, we see close agreement between the average estimate and the limiting value, and very close agreement between the average model-based and average robust standard errors. The empirical coverage probability (for α*) is also consistent with the nominal level, as is the empirical power.

#### 2.3.3 Composite endpoints with independent components

Table 2 presents the simulation results with independent components (i.e., τ = 0). The results in the top half of Table 2 reveal that the limiting value α* is the same as the common value β = β_1_ = β_2_ as expected because assumption A.1 of Section 2 is satisfied. Again, the average point estimate is in close agreement with this common value, and the three standard errors are in close agreement. When the treatment has an effect on *T*_1_ and not *T*_2_, α* is again considerably smaller than β_1_. Note, however, even though this is a misspecified model, the limiting value does not depend on the censoring distribution. This much smaller value leads to larger sample size requirements than in the top half of the table. Because the first component *T*_1_ happens less frequently than the second component *T*_2_ (i.e., *P*(*T*_1_ < *T*_2_|*Z* = 0) = 0.25), the limiting value from the misspecified Cox model is heavily attenuated in this setting. However, neither administrative nor random censoring appears to affect the limiting value of the estimator of treatment effect.

**Table 2:** Frequency properties of estimators of treatment effect based on a composite endpoint with independent components: *p*_1_ = *P*(*T*_1_ < *T*_2_|*z* = 0) = 0.25, β_1_ = − 0.223.

*π*_A_	*π*	*m*	α^*^	AVE( 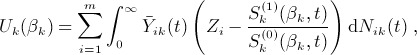 )	ESE	ASE_1_	ASE_2_	ECP^*^%	ECP%	EP%
			*Common treatment effect: β*_2_ = −0.223										
0.2	0.2	644	−0.223	−0.224	0.090	0.090	0.090	95.6	95.6	79.5
	0.4	865	−0.223	−0.225	0.090	0.090	0.090	95.0	95.0	80.6
	0.6	1310	−0.223	−0.227	0.090	0.090	0.090	95.3	95.3	80.7
	0.8	2654	−0.223	−0.223	0.088	0.090	0.090	95.6	95.6	80.4
0.4	0.4	872	−0.223	−0.226	0.089	0.090	0.090	95.6	95.6	81.5
	0.6	1315	−0.223	−0.226	0.090	0.090	0.090	95.8	95.8	80.3
	0.8	2655	−0.223	−0.223	0.088	0.090	0.090	95.2	95.2	80.6
0.6	0.6	1323	−0.223	−0.223	0.091	0.090	0.090	95.1	95.1	79.9
	0.8	2660	−0.223	−0.223	0.088	0.090	0.090	95.3	95.3	80.4
0.8	0.8	2670	−0.223	−0.221	0.091	0.090	0.090	94.8	94.8	78.5
			*Different treatment effects: β*_2_ = 0										
0.2	0.2	11,750	−0.051	−0.052	0.021	0.021	0.021	94.4	0.0	80.6
	0.4	15,666	−0.051	−0.052	0.021	0.021	0.021	94.9	0.0	81.0
	0.6	23,499	−0.051	−0.052	0.021	0.021	0.021	94.7	0.0	80.3
	0.8	46,998	−0.051	−0.052	0.020	0.021	0.021	95.6	0.0	81.2
0.4	0.4	15,666	−0.051	−0.052	0.021	0.021	0.021	95.2	0.0	81.1
	0.6	23,499	−0.051	−0.052	0.021	0.021	0.021	95.3	0.0	80.1
	0.8	46,998	−0.051	−0.052	0.020	0.021	0.021	95.3	0.0	81.3
0.6	0.6	23,500	−0.051	−0.052	0.021	0.021	0.021	94.1	0.0	81.5
	0.8	46,998	−0.051	−0.052	0.020	0.021	0.021	95.6	0.0	81.4
0.8	0.8	46,999	−0.051	−0.051	0.021	0.021	0.021	94.7	0.0	80.6

*π*_A_ = *P*(*C*^†^ < *T*) is the administrative censoring rate, *π* = *P*(*C*^†^ < *T*) is the net censoring rate, ESE is the empirical standard error, ASE_1_ is the average model-based standard error, ASE_2_ is the average robust standard error, ECP^*^% is the empirical coverage probability for α^*^ of nominal 95% CIs using the robust standard error, ECP% is the empirical coverage probability for β_1_ of nominal 95% CIs using the robust standard error, and EP% is the empirical power of a Wald test of *H*_0_ : *α* = 0 based on the robust standard error.

## 3 A multivariate semiparametric analysis

### 3.1 Limiting values for a Wei–Lin–Weissfeld analysis

In this section, we investigate the utility of the marginal approach of Wei *et al.* 1989 for handling multivariate failure time data. This approach is based on formulating ordinary Cox models for each component event to obtain component-specific estimates of treatment effect, and it is therefore compatible with the way the joint distributions were constructed using copula functions in Section 2. Estimation proceeds under a working independence assumption, as often adopted for analyses based on generalized estimating equations. We obtain a robust estimate of the covariance matrix, and then we obtain a global estimate of treatment effect by taking a weighted average of all component-specific estimates with weights chosen to minimize the variance of the global estimator. A key distinction between the global approach of Wei *et al.* 1989 and the composite endpoint approach is that the former makes use of all observed events whereas the composite endpoint uses only information on the first event.

In the derivations that follow, the composite endpoint is comprised of *K* components, but we subsequently focus on the case *K* = 2. We let *dN*_*ik*_(*s*) = *I*(*T*_*ik*_ = *s*), {*N*_*ik*_(*s*),0 < *s*} denote the counting process for type *k* events, and {*N*_*i*_(*s*) = (*N*_*i*1_(*s*),*N*_*i*2_(*s*),0 < *s*} denote the bivariate counting process for subject *i*, *i* = 1, …,*m*. Let *Y*_*ik*_(*s*) = *I*(*s ≤ T*_*ik*_), 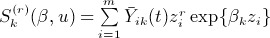, and 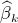, *k* = 1, …,*K*, *i* = 1, …,*m*. The Cox model for the type *k* event is 

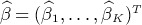
 where λ_*k*0_(*t*) is the baseline hazard function and β_*k*_ is the corresponding treatment effect. The *k*th component-specific score function for β_*k*_ is 

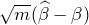
 where 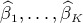, *r* = 0,1.

Under the copula model of Section 2 with marginal distributions featuring proportional hazards, the solution to the score equation 24, 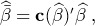, is consistent for the true treatment effect β_*k*_. If we let β = (β_1_, …,β_*K*_)′ and its estimate 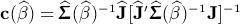, Wei *et al.* 1989 show that 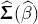 converges in distribution to a multivariate normal distribution with a zero-mean vector and variance–covariance matrix ***Σ***(β) and provides a consistent sandwich-type estimate for ***Σ***(**β**).

The global estimate of treatment effect proposed by Wei *et al.* 1989 is a linear combination of all component-specific treatment effect estimates 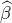 and can be obtained as 

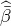
 where the weight 
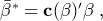
 is chosen to estimate the weight matrix to minimize the variance in the class of all linear estimators; 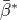 is the estimate for the variance–covariance matrix of 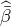 and **J** = (1, …,1) ′ .

To compare the performances of the global approach and the composite endpoint approach, we obtain the limiting value of 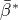 as 

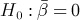
 where **c**(β) = ***Σ***^−1^(β)**J**[**J** ′ ***Σ*** − 1(β)**J**]^−1^. We therefore require the limiting value of the robust variance ***Σ***(β) to obtain the limiting value. The detailed derivations are deferred to the Appendix.

An alternative asymptotically equivalent approach to estimating the global effect and to deriving the limiting value involves specifying a single Cox regression model and fitting it using all events while ‘stratifying’ on the event type [32]. Although this has some appeal, we adopt the current framework on the basis of synthesizing estimates from separate Cox regression models because it makes explicit the fact that the global estimate and associated limiting value may be viewed as a weighted average of the component-specific estimates.

### 3.2 Comparison of the global approach and the composite endpoint analysis

Table 3 reports the results from a global analysis of treatment effect based on the marginal analysis proposed by Wei *et al.* 1989. In this table, the sample sizes were computed on the basis of the formula for the composite endpoint analysis using the limiting value of the regression coefficient. As one would expect from 24, when the treatment effects are equal, then the marginal analysis yields consistent estimators for this common effect and the mean estimate across all simulated trials is very close to the limiting value. Moreover, the ESE and the average robust standard error were in very close agreement; the average model-based standard error is conservative because it is based on the working independence assumption being correct. The empirical coverage probabilities (based on the robust standard errors) were compatible with the nominal 95% level for 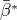 when β_1_ = β_2_. When β_1_ ≠ β_2_, the empirical coverage for β_1_ was zero, a reflection of the difference between 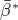 and β_1_. When β_2_ = 0, the limiting value 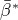 was quite small, and hence, the sample sizes of the trial were much larger.

**Table 3:** Empirical properties of the global estimates of treatment effect based on Wei–Lin–Weissfeld analysis: data were generated under a Clayton copula with τ = 0.40, β_1_ = −0.223.

*π*_A_	*π*	*m*	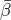	AVE( 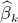 )	ESE	ASE_1_	ASE_2_	ECP^*^%	ECP%	EP%
			*Common treatment effect: β*_2_ = −0.223										
0.2	0.2	621	−0.223	−0.223	0.084	0.072	0.086	95.9	95.9	83.6
	0.4	828	−0.223	−0.223	0.086	0.074	0.087	95.1	95.1	82.0
	0.6	1242	−0.223	−0.221	0.088	0.077	0.088	95.0	95.0	80.8
	0.8	2484	−0.223	−0.223	0.089	0.083	0.090	95.6	95.6	80.3
0.4	0.4	828	−0.223	−0.223	0.087	0.076	0.087	95.4	95.4	82.7
	0.6	1242	−0.223	−0.221	0.089	0.078	0.088	95.0	95.0	79.9
	0.8	2484	−0.223	−0.223	0.089	0.083	0.090	95.6	95.6	80.6
0.6	0.6	1242	−0.223	−0.223	0.090	0.081	0.089	95.1	95.1	79.7
	0.8	2484	−0.223	−0.222	0.089	0.083	0.090	95.2	95.2	80.5
0.8	0.8	2484	−0.223	−0.225	0.088	0.086	0.090	95.2	95.2	80.5
			*Different treatment effects: β*_2_ = 0										
0.2	0.2	7090	−0.066	−0.067	0.025	0.021	0.025	95.9	0.0	84.2
	0.4	9664	−0.065	−0.066	0.025	0.022	0.025	94.5	0.0	83.3
	0.6	14623	−0.065	−0.066	0.026	0.023	0.026	94.8	0.0	82.8
	0.8	28219	−0.066	−0.066	0.026	0.024	0.027	95.3	0.0	81.7
0.4	0.4	10203	−0.064	−0.065	0.025	0.022	0.025	95.1	0.0	83.6
	0.6	14897	−0.064	−0.066	0.025	0.023	0.025	94.6	0.0	83.2
	0.8	28316	−0.066	−0.066	0.026	0.024	0.027	95.2	0.0	80.6
0.6	0.6	14733	−0.065	−0.066	0.026	0.024	0.026	94.1	0.0	83.4
	0.8	28202	−0.066	−0.067	0.026	0.025	0.027	95.2	0.0	81.7
0.8	0.8	27355	−0.067	−0.069	0.026	0.026	0.027	95.4	0.0	82.2

*π*_A_ = *P*(*C*^†^ < *T*) is the administrative censoring rate, *π* = *P*(*C* < *T*) is the net censoring rate, ESE is the empirical standard error, ASE_1_ is the average model-based standard error, ASE_2_ is the average robust standard error, ECP^*^% is the empirical coverage probability for 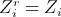 of nominal 95% CIs using the robust standard error, ECP% is the empirical coverage probability for β_1_ of nominal 95% CIs using the robust standard error, and EP% is the empirical power of a Wald test of 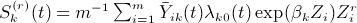 based on the robust standard error.

**Figure 3:**
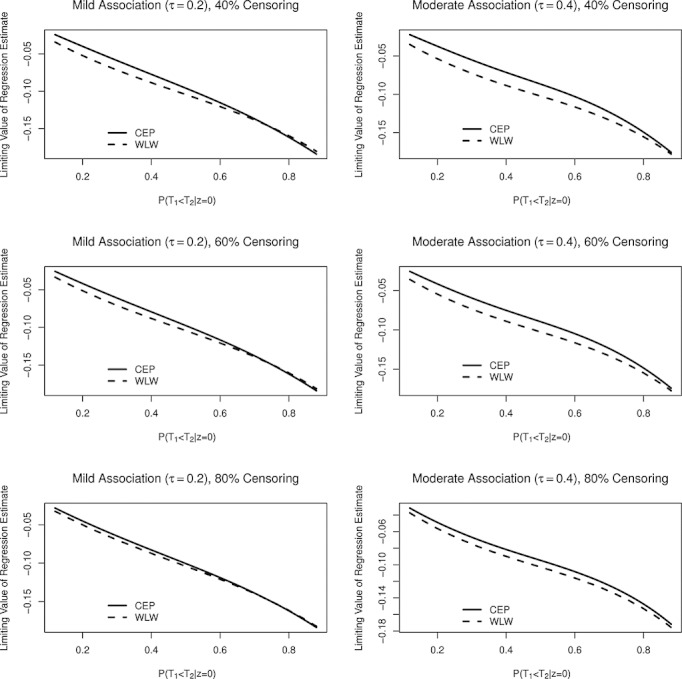
Plot of limiting values of regression estimates of treatment effect based on a composite endpoint analysis and a global Wei *et al.* 1989 analysis with bivariate data generated with a Clayton copula; β_1_ = log 0.80, β_2_ = 0.

When β_1_ ≠ β_2_, the composite endpoint and global analyses yield estimators that do not coincide with β_1_, β_2_, or each other. We next compare these limiting values. We consider the case in which two failure times are generated by a Clayton copula with exponential margins and a single treatment covariate modeled through proportional hazards with β_1_ = log(0.80) and β_2_ = 0. We consider mild and moderate association between the failure times with τ = 0.20 and τ = 0.40, respectively. Administrative censoring was set to 40% and additional random censoring from an exponential withdrawal time gave cases with 60% and 80% as well. The limiting values of the composite endpoint and global analyses were plotted against *P*(*T*_1_ < *T*_2_|*Z* = 0) = *p*_1_ in Figure 3. It is apparent that when *p*_1_ approaches zero, the limiting value for both methods approaches 0. For the composite endpoint, this makes sense because the first event is most likely to be a type 2 event for which there is no treatment benefit. As *p*_1_ approaches 1, the limiting value for the composite endpoint analysis approaches β_1_ for analogous reasons. The limiting value from the global analyses tracks these limiting values quite well, but tend to correspond to larger estimates of treatment effect because the limiting value is larger in absolute value. Thus, even when the two components have equal frequencies and the proportional hazards assumption holds for each component, the global analysis, in the limit, will yield an estimate of treatment effect that is greater than that of the composite endpoint analysis. These relationships hold across both levels of association and over different degrees of censoring. Although we have restricted attention to the Clayton copula in these calculations and empirical studies, this investigation could be repeated under other copula models, and although the limiting values would differ, qualitatively similar findings would be expected.

## 4 Application to an asthma management study

We now apply both the composite endpoint analysis and the global approach to an asthma management study [33]. This is a two-phase, multicenter, randomized, parallel group effectiveness trial for comparing two treatment strategies for asthma management over a 2-year period. The control strategy is a ‘clinical strategy’, in which the treatment was guided on the basis of patient symptoms and spirometry readings. The experimental strategy is a so-called ‘sputum strategy’ (SS), whereby a cellular analysis of sputum samples was used to guide corticosteroid therapy use to keep eosinophils cell counts less than 2%. In phase I, a total of 107 patients were identified through the minimum treatment to maintain control. The aim of this asthma study was to investigate whether SS is more effective than clinical strategy on reducing the number and severity of exacerbations in phase II.

**Figure 4:**
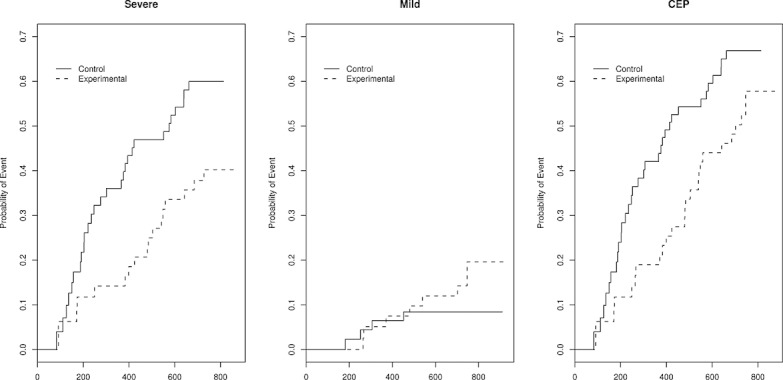
Empirical distribution functions for severe exacerbations, mild exacerbations, and the composite endpoint in asthma trial.

In our analysis, we focus on two types of exacerbations: mild exacerbations defined as requiring a daily maintenance dose of fluticasone of < 250 μg and severe exacerbations defined here as requiring a minimum daily maintenance dose ≥250 μg. The composite endpoint is defined as the time to the first of the two type of exacerbations. Figure 4 displays the empirical distribution function plots for the two component types of exacerbations and for the composite endpoint. It is apparent that the severe exacerbations occur much more frequently than mild exacerbations and thus represent the majority of the events contributing to the composite endpoint.

Table 4 presents the results of the proportional hazards regression analysis in which the single binary covariate is the treatment indicator taking the value one for patients in the experimental (SS) group and zero otherwise. From these results, it is clear that the experimental SS strategy leads to a significantly lower hazard of severe exacerbations with a relative risk reduction of 47% (95% CI: 0.02, 0.71; *p* = 0.042) but has little effect on the occurrence of mild exacerbations (*p* = 0.227). The result from the composite endpoint analysis is not statistically significant with *p* = 0.137. The Wei–Lin–Weissfeld 1989 global analysis yields an estimate that is close to that obtained from the composite endpoint analysis, but it is apparent when examining the effects on the separate components that a global estimate is not an adequate summary of the data. The last column of Table 4 gives the *p*-values for testing the proportional hazards assumption using univariate tests based on Schoenfeld residuals [32]. There is insufficient evidence to reject the null hypothesis of proportional hazards for each component, and the test yields a *p*-value just shy of statistical significance for the composite endpoint analysis at 0.063. Thus, although we have demonstrated that, in principle, if the proportional hazards assumption holds for the components of a composite endpoint, it generally does not hold for composite endpoint itself, the tests do not suggest problems with model fit for this particular data. Although the association may be well characterized by the Gumbel–Hougaard copula, the power of the tests for departures from proportional hazards may also be inadequate.

**Table 4:** Analysis results of the asthma management study.

Endpoint/Analysis	RR	95% CI	*p*-value	p^*^
Severe	0.53	(0.285, 0.977)	0.042	0.22
Mild	2.14	(0.624, 7.310)	0.227	0.11

Composite	0.665	(0.388, 1.138)	0.137	0.063

Global (WLW)	0.702	(0.405, 1.219)	0.209	

## 5 Discussion

Composite endpoints are widely adopted in clinical trials, and fitting a Cox proportional hazards model is the standard approach to estimating treatment effects on the basis of such endpoints. We have demonstrated that even when the treatment effects are the same for component endpoints under marginal Cox models, the Cox model for the composite endpoint is typically misspecified because the proportional hazards assumption does not in general hold. The estimator of treatment effect under such a misspecified Cox model for the composite endpoint has a slightly conservative limiting value, meaning that the benefit of treatment was under-estimated in the settings we examined. We found several factors that influence the limiting value including the strength of the association between the individual component events, stochastic ordering of the individual components, and the degree and nature of the censoring process; empirical studies corroborated these findings. Although we have not explored this here, it is clear from Section 2 that the specific copula function would also have an important effect. More generally, variation in the treatment effect across the individual components makes it even more difficult to interpret estimators.

Composite endpoints are often thought to offer a measure of the ‘overall effect’ of a treatment [9]. In fact, the opposite can be true if treatment effects are in opposing directions for different components, and one component tends to occur first. The event tending to occur first will have the greatest influence on the estimator of treatment effect based on the composite endpoint, masking the effect on the events that tend to occur later. In the case where the events occur with equal frequency and the treatment effect is the same for the components, the asymptotic calculations of Section 2 show that the estimate based on the composite endpoint suggests a smaller benefit that holds for the components. Although some might argue that this is therefore a conservative approach, it is essentially incorrect. The global approach of Wei *et al.* 1989 can, however, provide evidence of this adverse effect in the component-wise analysis, and this will lead to an attenuation of the global effect in the weighted analysis.

Another rationale put forward for adopting composite endpoints is to model the event-free survival probability. For example, Sheehe 2010 proposed that the event-free survival curve can be computed on the basis of Cox model estimates of hazard ratios from the composite endpoint containing mortality as a component. As we have demonstrated, estimators from Cox regression using composite endpoints can be attenuated by including components for which there is no treatment effect. Using estimates from the composite endpoint analysis (event-free survival) may not provide a valid representation of the effect on the non-fatal event.

Two of the guidelines for the use of composite endpoints include the requirement that individual component events should be of roughly equal frequency, and the treatment effects should be comparable across all components. Our analytical and empirical investigation shows that these may not be sufficient conditions if interest lies in estimating these common effects in the sense that even when these conditions are satisfied, the association between the two events can lead to substantial bias in estimators based on composite endpoints. We support the recommendations that (i) data from all components should be followed until the end of the trial and (ii) individual components should be analyzed and reported separately. This alternative design and analysis facilitates a global approach [20] based on combining estimates from individual components, as well as assessment of whether this is appropriate. In the context of the copula-based joint model, we found that the global approach, in general, outperforms the composite endpoint analysis in terms of the properties of the resulting estimators and power or sample size requirements.

We have formulated a model with proportional hazards for each component event through the use of a copula function to reflect an idealized situation in alignment with the recommendations in the literature. We restricted attention to the situation with two component endpoints, but three or more components are often specified in practice. When multiple components are of interest, copula functions with an ‘exchangeable’ association structure can be readily adopted; more baseline marginal hazard functions and treatment effects would need to be specified. It is relatively straightforward to extend the derivations and empirical studies reported here for this setting but more challenging to cover a meaningful spectrum of settings, summarize results, and make recommendations.

Alternative frameworks could naturally be adopted for specifying models for correlated failure time data. One might, for example, consider intensity-based models where the risk of one type of event changes with the occurrence of another type of event. This could arise because of a biological mechanism in which the medical risk actually increases or if treating physicians alter the therapy being given. This formulation, although natural for characterizing the response process, is not compatible with proportional hazards for the marginal models. One might also consider frailty models for addressing the association between event times, but again, the marginal models will not have a proportional hazards form.

We have assumed independent censoring in this paper. Another way in which patients may be treated differently following the occurrence of a clinically important event is to be withdrawn from a study. The occurrence of one event may increase the risk an investigator may withdraw the patient from the study and result in response-dependent censoring. If the events are independent conditional on the treatment covariate, this will not pose a problem but otherwise will lead to biased estimates of the baseline hazard functions and treatment effects. Use of inverse probability of censoring weights will help reduce this bias, and this is currently under investigation.

Finally, we have focused on the frequency properties of estimators under a Cox regression models. Cox models are used routinely, but of course, the proportional hazards assumption may not be valid for either the marginal distributions or the composite endpoint. There is increasing interest in use of alternative regression models for the analysis of survival data including accelerated failure time models and additive models. Exploration of the behavior of estimators from such models warrants study.

## Appendix

Derivation of the limiting value

Suppose the proportional hazards assumption holds for the marginal distribution of each component event and a copula model is used to characterize the association. Under a working independence assumption [20], the limiting value for  is β_*k*_. Here, we consider a generic scalar covariate *Z*_*i*_, but note that the functions simplify with *Z*_*i*_ a binary treatment indicator because  in this case. Let , , , and , *r* = 0,1,2, where *E*(·) denotes expectation with respect to the true distribution. Let , *k* = 1, …,*K*, where the *k*th diagonal element of  is

by the Theorem 4.2 of Andersen and Gill 1982. In the present setting, the true model is known, the required expectations can be obtained in closed form, and the integral can be evaluated using numerical methods.

If

is the martingale for events of type *k*; let

and **w**_*i*_(β) = (*w*_*i*1_(β_1_), …,*w*_*iK*_(β_*K*_))′. Then if we define , the asymptotic robust covariance matrix ***Σ***(β) takes the form [20]. This can be used to obtain the limiting value through 26.

The entries of  are obtained as follows. The (*j*,*j*) element of  is

where 〈·, ·〉 is the predictable covariation process and the last equality holds because of Fubini's theorem [36]. The (*j*,*k*) element of  is then

Usf Prentice and Cai 1992, 〈d*M*_*j*_(*t*_*j*_),d*M*_*k*_(*t*_*k*_)〉 can be obtained. In the case of bivariate data, 〈d*M*_1_(*t*_1_), d*M*_2_(*t*_2_)〉 is obtained simply as

where Λ_*k*_(d*t*_*k*_|*z*_*i*_; Ω) = dΛ_*k*_(*t*_*k*_|*z*_*i*_; Ω) / d*t*_*k*_; , , and . More specifically, if the joint survivor function  is specified by the Clayton copula with margins of two exponential distributions, then 〈d*M*_*j*_(*t*_*j*_),d*M*_*k*_(*t*_*k*_)〉 can be obtained in closed form and *E*(*w*_*ij*_(β_*j*_)*w*_*ik*_(β_*k*_)) can be obtained through numerical integration. Thus, we obtain the limiting value of robust variance, then the limiting weights can be calculated using **c**(β) = ***Σ***^−1^(β) / **J** ′ ***Σ*** − 1**J** and the limiting value  using equation 12.
